# Assessment of Neuroprotective Effects of Low-Intensity Transcranial Ultrasound Stimulation in a Parkinson’s Disease Rat Model by Fractional Anisotropy and Relaxation Time T2^∗^ Value

**DOI:** 10.3389/fnins.2021.590354

**Published:** 2021-02-09

**Authors:** Yanchao Dong, Defeng Liu, Yuemei Zhao, Yi Yuan, Wenxi Wang, Shuo Wu, Xin Liang, Zhanqiu Wang, Lanxiang Liu

**Affiliations:** ^1^Department of Interventional Treatment, Qinhuangdao Municipal No. 1 Hospital, Qinhuangdao, China; ^2^Department of Magnetic Resonance Imaging, Qinhuangdao Municipal No. 1 Hospital, Qinhuangdao, China; ^3^College of Electrical Engineering, Yanshan University, Qinhuangdao, China

**Keywords:** Low-intensity transcranial ultrasound, Parkinson’s disease, magnetic resonance imaging, diffusion tensor imaging, substantia nigra, neuroprotection, Fractional anisotropy (FA), relaxation time T2^∗^

## Abstract

**Background:** Low-intensity transcranial ultrasound (LITUS) may have a therapeutic effect on Parkinson’s disease (PD) patients to some extent. Fractional anisotropy (FA) and relaxation time T2^∗^ that indicate the integrity of fiber tracts and iron concentrations in brain tissue have been used to evaluate the therapeutic effects of LITUS.

**Purpose:** This study aims to use FA and T2^∗^ values to evaluate the therapeutic effects of LITUS in a PD rat model.

**Materials and Methods:** Twenty Sprague-Dawley rats were randomly divided into a hemi-PD group (*n* = 10) and a LITUS group (*n* = 10). Single-shot spin echo echo-planar imaging and fast low-angle shot T2WI sequences at 3.0 T were used. The FA and T2^∗^ values on the right side of the substantia nigra (SN) pars compacta were measured to evaluate the therapeutic effect of LITUS in the rats.

**Results:** One week after PD-like signs were induced in the rats, the FA value in the LITUS group was significantly larger compared with the PD group (0.214 ± 0.027 vs. 0.340 ± 0.032, *t* = 2.864, *P* = 0.011). At the 5th and 6th weeks, the FA values in the LITUS group were significantly smaller compared with the PD group (5th week: 0.290 ± 0.037 vs. 0.405 ± 0.027, *t* = 2.385, *P* = 0.030; 6th week: 0.299 ± 0.021 vs. 0.525 ± 0.028, *t* = 6.620, *P* < 0.0001). In the 5th and 6th weeks, the T2^∗^ values in the injected right SN of the LITUS group were significantly higher compared with the PD group (5th week, 12.169 ± 0.826 in the LITUS group vs. 7.550 ± 0.824 in the PD group; 6th week, 11.749 ± 0.615 in the LITUS group vs. 7.550 ± 0.849 in the PD group).

**Conclusion:** LITUS had neuroprotective effects and can reduce the damage of 6-OHDA-induced neurotoxicity in hemi-PD rats. The combination of FA and T2^∗^ assessments can potentially serve as a new and effective method to evaluate the therapeutic effects of LITUS.

## Introduction

Parkinson’s disease (PD) is a neurodegenerative disease that frequently occurs in older adults (Sy and Fernandez, 2020; Zhao F. et al., 2020; Zhao P. et al., 2020). The classic clinical manifestations of PD include bradykinesia, resting tremor, rigidity, and postural instability. These signs are caused primarily by the loss of dopamine (DA) in the striatum due to the progressive death or impairment of dopaminergic neurons in the substantia nigra (SN) pars compacta. For all patients with PD, treatment is symptomatic, focused on improvement in motor and non-motor signs and symptoms (Armstrong and Okun, 2020). Dopamine-based therapies typically help initial motor symptoms. Non-motor symptoms require non-dopaminergic approaches (e.g., selective serotonin reuptake inhibitors for psychiatric symptoms, cholinesterase inhibitors for cognition) (Ryan et al., 2019). Rehabilitative therapy and exercise complement pharmacologic treatments (Carroll et al., 2017). Palliative care is part of Parkinson’s disease management (Armstrong and Okun, 2020). The mainstay of PD treatment is pharmacological, primarily targeted at increasing dopaminergic activity in the nigrostriatal pathway (Armstrong and Okun, 2020). However, as the disease progresses, increasing doses of drugs are required, which are associated with the onset of unwanted drug-induced dyskinesias and motor fluctuations (Antonini and Nitu, 2018). Therefore, new treatments are needed that are non-invasive and with few to no side effects.

Previous studies have demonstrated that low-intensity transcranial ultrasound stimulation (LITUS) can be used as a new modality for non-invasive neuromodulation, and the technology is developing rapidly (Naor et al., 2016; Landhuis, 2017). Compared with transcranial magnetic stimulation and deep brain stimulation, LITUS has the advantages of being highly focused, safe, and effective for neuroprotection (Liu et al., 2019). Previous studies have demonstrated that LITUS can induce neural responses *in vitro*, promote protein expression (Liu S.H. et al., 2017), and modulate brain activity (Yuan et al., 2019; Wang et al., 2020), hemodynamics, and oxygen metabolism (Kim et al., 2017). Studies have verified that LITUS can inhibit neuronal firing rhythms that occur during epilepsy and improve symptoms in consciousness disorders after severe brain injury (Hakimova et al., 2015). Currently, researchers have used ultrasound brain stimulation to treat PD mouse successfully. The research found that ultrasound brain stimulation enables modulation of neural activity and leads to neuroprotection in PD mice and is a noninvasive strategy for the treatment of PD (Zhou et al., 2019). At present, few scholars have studied the protective mechanism of LITUS on the brain nerve of hemi-PD rats.

Some studies have reported that diffusion tensor imaging (DTI) and T2^∗^WI could also monitor the therapeutic effects of drugs (Liu L.X. et al., 2017; Fang et al., 2018). The value of FA can reflect the complexity of neural structure in the brain. The more complex the neural structure, the higher the FA (Siasios et al., 2016). In addition, the T2^∗^ value reflects the deposition of iron ions in the brain. The more iron ions are deposited in the brain, the lower the T2^∗^ value (Costa-Mallen et al., 2017). Specifically, the FA values of hemi-PD rats were significantly higher, and the T2^∗^ values of hemi-PD rats were significantly lower compared with untreated rats (Fang et al., 2018). However, neither DTI nor T2^∗^WI were used to monitor hemi-PD rats weekly, and neither technique was used to evaluate the therapeutic effect of LITUS in detail. In this study, DTI and T2^∗^WI were used to monitor the pathological changes occurring in the SN in PD rats induced by 6-OHDA. We also evaluated the therapeutic effects of LITUS on hemi-PD rats. Our goal was to provide a new therapeutic measure that could benefit the PD patients and provide an evaluation method that could assess the curative effect of LITUS for the PD treatment.

## Materials and Methods

### Animals and Surgery

**FIGURE 1:**
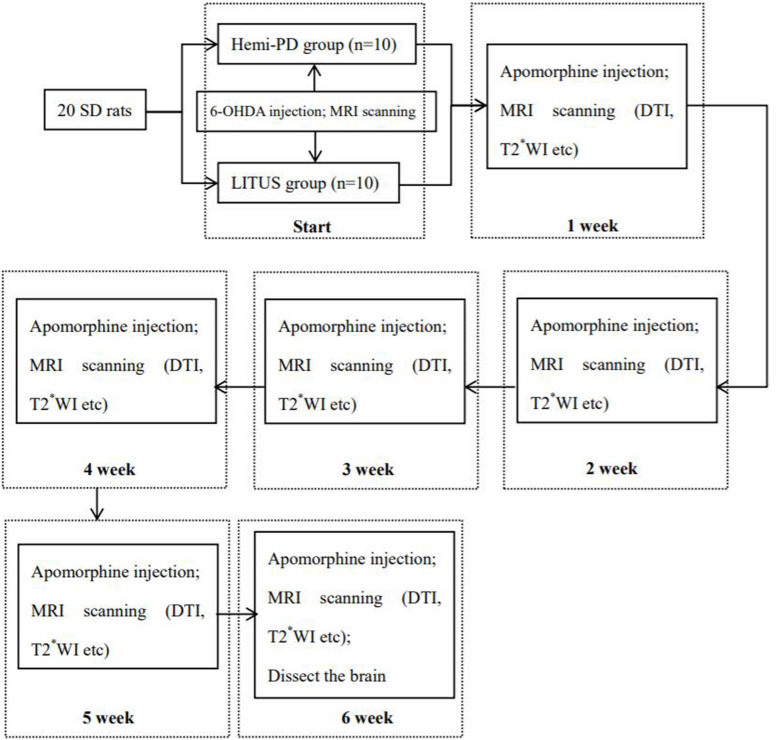
The timeline of this experiment.

All experiments were performed on mature (7-week-old; 200–250 g) male, Sprague-Dawley rats (*n* = 20) (Beijing Vital River Laboratory Animal Technology Co., Ltd. China). The experiments were conducted in accordance with national guidelines for the use of experimental animals. All experimental protocols were approved by the Ethics Committee of Qinhuangdao Municipal No. 1 Hospital (201802B005, 2018-12-15). The rats were housed in a temperature- (22 ± 2°C) and humidity-controlled (60 ± 5%) room, on a 12-h light/dark cycle, and with access to food and water *ad libitum*. Rats were randomly divided into two groups: (1) a hemi-PD group (*n* = 10) and (2) a LITUS group (*n* = 10). The surgical procedures and rotation test that were used for induction and assessment of the PD model followed the published protocols by Fang et al.; all 20 rats were subjected to the modeling process (Fang et al., 2017). First, the rat was anesthetized using 10% chloral hydrate by intraperitoneal injection, initial measurement was 3 ml/kg, and maintained dose was 2 ml/kg/h for the duration of the operation. The rat was mounted into a stereotactic apparatus (Lab Standard Stereotaxic-Single, Stoelting Co, Illinois, United States), bregma was exposed, an incision was made on the scalp along the sagittal suture, and a small trephine hole was drilled in accordance with the stereotactic coordinates of the right SN (relative to bregma: AP, −4.8 mm; ML, 1.9 mm; DV, −8.0 mm from skull). We chose coordinate points according to George Paxinos’ book the *Rat Brain in Stereotaxic Coordinates, Compact, Third Edition*. Using a microsyringe (Hamilton Bonaduz AG, Bonaduz, Switzerland), 6 μl of 6-OHDA solution (2 μg/μl in normal saline containing 0.2% ascorbate; Sigma Chemical Co., St. Louis, MO, United States) was automatically infused into the right SN of rats in the two groups. Although drugs can also cause serious damage to noradrenergic neurons, neither did we protect noradrenergic neurons before injecting 6-OHDA (Lindgren et al., 2014); it was because we considered taking measures to protect noradrenergic neurons in rats, which may increase the burden on rats. In addition, we considered that the loss of noradrenergic nerve by 6-OHDA has little effect on the experimental results. The needle was kept in place for 5 min after completion of the injection and then slowly withdrawn. The scalp was sutured closed, and intramuscular antibiotics were administered to prevent infection. See Figure 1 for the timeline of this experiment.

### Apomorphine-Induce Rotation Test

All rats received a single intraperitoneal injection of apomorphine (0.5 mg/kg in normal saline) at the beginning of every week after surgery. For rotation test, animals were allowed to habituate to the test apparatus for 10 min and then for an additional 2 min after the injection. Full rotations were counted in a cylindrical container in a dimly lit, quiet room. Rotational asymmetry was scored continuously for 30 min and then complete contralateral rotation times were scored. Rats with test scores greater than 7 were retained for the study and analysis (Fang et al., 2017).

After 6-OHDA lesion, we needed to carry out rotation test to verify the success of the animal model. At present, it was generally believed that the rat model was successfully prepared if the rotation experiment exceeds seven times per minute.

### LITUS Protocol

A commercial ultrasonic brain stimulator (DK-102T, Dukang Medical Devices, Co., Ltd., Shijiazhuang, China) was used to produce the transcranial ultrasound used in the experiments. The area of the unfocused ultrasound transducer was 3.5 cm^2^. The total stimulation duration was 10 min, with a total of 200 trials. The ultrasound fundamental frequency (FF) and pulse-repetition frequency (PRF) were 500 and 1 kHz, respectively. The ultrasound stimulation duration (SD) and tone-burst duration (TBD) parameters were 400 and 0.5 ms, respectively. The ultrasound pressure was measured using a calibrated needle-type hydrophone (HNR500, Onda, Sunnyvale, CA, United States), and the spatial peak and pulse-average intensity (Isppa) was 2.6 W/cm^2^ (Liu et al., 2019). The LITUS group rats underwent ultrasonic stimulation every day after the surgical procedure (Liu et al., 2019). The stimulation site was the same side of the brain as the drug injection site.

### MR Imaging

**FIGURE 2:**
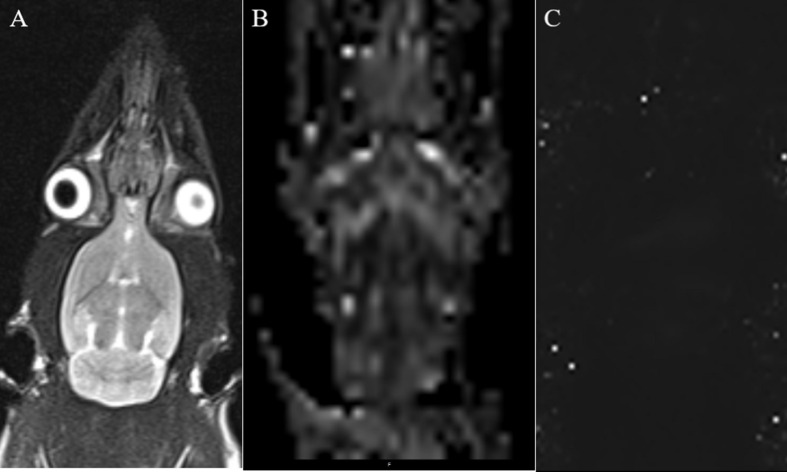
**(A)** Coronal T2WI—we used it as a reference image when measuring values. **(B)** Coronal black and white FA value image for measuring FA value in SN area. **(C)** Coronal T2 maps image for measuring T2* value in SN area.

Anesthesia was performed as described above, and the rats were scanned using a 3.0-T MRI system (Verio, Siemens Medical Solutions, Erlangen, Germany) with an eight-channel coil. The brain images were obtained using coronal T2WI turbo spin-echo (TSE). Single-shot spin echo echo-planar imaging (SE-EPI) was used in DTI, and a fast low-angle shot 2D T2WI (FLASH 2D T2WI) was used to acquire the T2 maps, based on the parameters specified by Liu (Fang et al., 2018). The rat’s head was immobilized with a custom-made MRI-compatible head holder. The image quality was analyzed immediately after the MR scan was completed. If the rat’s head moved during the MR scan, it was repeated 2 h later with an additional anesthetic as needed. Imaging analysis was carried out using prototype software on a workstation (Siemens Verio 3.0 T MR Leonardo 3682). Figure 2 shows the coronal MRI of rat brain. Using T2WI image as a reference, the SN area can be identified on the FA and T2^∗^ parameter maps image, and the above scanning parameters were used to scan the SN area. The scanning time was the first, second, third, fourth, fifth, and sixth weeks after 6-OHDA lesion (Fang et al., 2018).

Two independent radiologists experienced in neural MRI analysis and who were blinded to the animal groups indicated regions of interest (ROIs) in the center of the SN on the right side of the brain that measured 0.30 to 0.60 cm^2^. The mean values of the repeated measurements taken in the SN were used in the final analysis.

### Immunohistochemistry and Histochemistry

Twenty rats were anesthetized again after MRI scans. The brains were removed, and the tissues were prepared as published previously. Coronal vibrate sections were cut from each brain and sliced into 8 μm sections through the ventral mesencephalon. The SN sections were selected for immunohistochemistry, and all the sections from different rat brains were matched as closely as possible (Dong et al., 2020). Immunohistochemistry for tyrosine hydroxylase (TH) and glial cell line-derived neurotrophic factor (GDNF) was performed by overnight incubation with the appropriate primary antibody (rabbit antirat; 1:300; Abcam, Cambridge, United Kingdom) at 4∘C. Sections were next incubated with secondary antibody for 30 min at 37∘C. Sections were visualized with 3,3’-diaminobenzidine, and nuclei were counterstained with hematoxylin and eosin (HE). Transverse frozen sections (5 μm) were dried in a baking box at 60∘C for 30–60 min and then soaked overnight in a 1:1 mixture of alcohol and chloroform in the dark at 22 ± 1∘C. Sections were rehydrated on the following day and stained with 0.1% cresyl violet solution (Sigma, St. Louis, MO, United States) for 5 min. Differentiation, dehydration, and rinsing were performed as described previously. Finally, sections were mounted with Permount (Beyotime Institute of Biotechnology, Shanghai, China) (Umbreen et al., 2017; Dong et al., 2020). Histochemical staining for iron hematoxylin was carried out on additional sections, as described by Zhang et al. (2014). Sections were processed through a series of graded alcohols, into xylene, and rehydrated back to water. Sections were incubated in a 1:1 solution of 2% HCl and potassium ferrocyanide (Sigma-Aldrich, St. Louis, MO, United States) for 30 min and rinsed in water. Sections were counterstained with Neutral Red, dehydrated in increasing concentrations of ethanol, cleared in xylene, and mounted on slides (Zhang et al., 2014).

The stained sections were scored according to the procedure described by Fang et al. (2018) with minor modifications. Twenty high-power fields were randomly chosen from each rat SN in the two groups. The number of positive cells and the total number of cells under high-power vision were calculated by naked eyes. The immunostaining was semiquantitatively expressed as follows: percentage of positive cells: 0, 0–10%; 1+, 11–25%; 2+, 26–50%; 3+, 51–75%; 4+, 76–100%; and intensity of immunostaining: 1+, weak staining; 2+, moderate staining; 3+, strong staining. The results of these two scoring methods were added to create the following grading scores: 0–2 represented weak expression, 3–5 represented moderately positive expression, and 6–7 represented strong positive expression. The mean values of the 20 high-power fields also were calculated, and the results were rounded to the nearest whole number.

### Statistical Analysis

Statistical analyses were performed using SPSS (Chicago, IL) version 21.0 software. Data were presented as the mean ± SD. The significance of the difference between the two groups was evaluated using a non-paired *t* test. Statistical differences were considered significant when *P* < 0.05.

## Results

### Comparison of FA and T2^∗^ Values Between the PD and LITUS Groups

All PD model rats scored more than 7 r/min with the rotation test; for detailed rotation test data of each rat, see Supplementary Table 1. We observed that the average FA value for the right SN of the hemi-PD rats in the first week was lower than the average FA value for the LITUS group. In the fifth and sixth weeks, the average FA value for the right SN of the hemi-PD rats was increased compared with the LITUS group, and the differences were statistically significant. From the second to fourth weeks, there was no significant difference in FA values between the two groups (see Supplementary Table 2 for details).

For the T2^∗^ values, in weeks 1, 2, 3, and 4, there were no significant differences in the T2^∗^ parameters observed for the right SN between the two groups (Fang et al., 2018). However, in the fifth and sixth weeks, the T2^∗^ values of the right SN for the LITUS group were significantly higher than those of the PD group, as shown in Supplementary Table 3.

**FIGURE 3:**
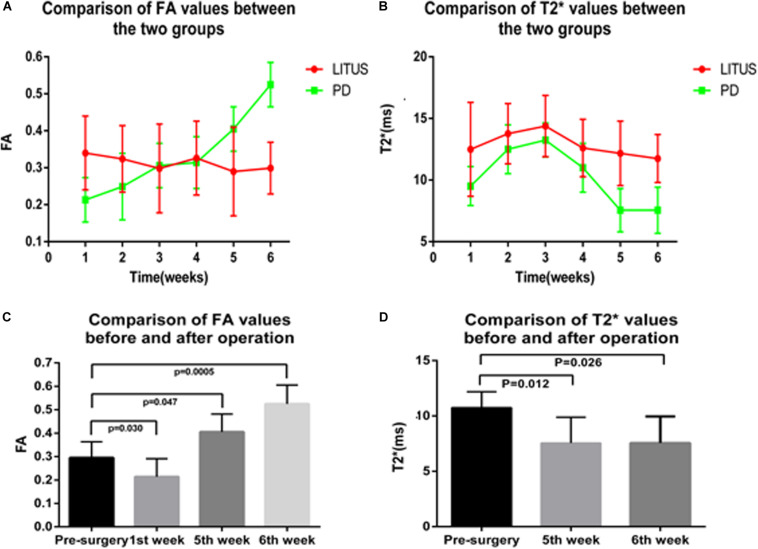
**(A,B)** The trend of FA and T2* values in the SN of the two groups. **(C,D)** The comparison results of FA and T2* values before and after modeling of the two groups.

As shown in Figure 3, the FA values in the right SN for the PD group revealed an upward trend, and the FA value increased from 0.295 ± 0.024 to 0.525 ± 0.028. However, the opposite trend was observed for the LITUS group, and the FA value decreased from 0.303 ± 0.016 to 0.299 ± 0.021. On the other hand, the trend of change in the T2^∗^ values was different in the two groups. The trend of change in the T2^∗^ values in the two groups was first increased and then decreased. The T2^∗^ values reached a peak in the third week, but the final pattern of decrease in the LITUS group was much less than the trend observed for the PD group.

### Comparison of the TH, GDNF, and Iron Staining in the Sixth Week

**FIGURE 4:**
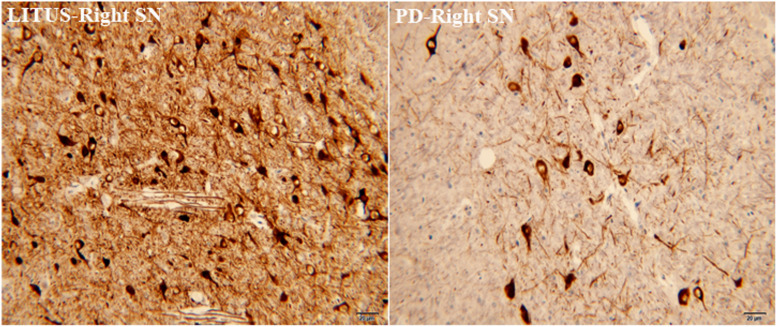
TH staining showed that the number of TH-positive cells in the PD group was significantly less than that in the LITUS group.

In the sixth week, after the 6-OHDA injection, there was distinct apoptosis of TH+-stained cells seen in the right SN in the hemi-PD rats. Furthermore, the damaged area in the hemi-PD rats was larger compared with the similar area in the LITUS-treated rats. The cell density was most noticeably reduced close to the injection point. The mean staining scores for the 10 rats were all between 0 and 2 in the hemi-PD rat group. However, for the LITUS-treated rats, the extent of neuronal loss in the side that received the LITUS treatment appeared to be less than in the hemi-PD rats. The mean staining scores for the LITUS rats were all between 4 and 5, as shown in Figure 4 and Supplementary Table 4.

**FIGURE 5:**
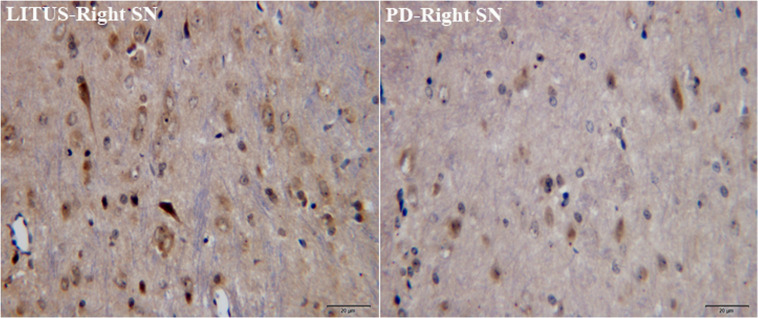
GDNF staining showed that the number of GDNF+ cells in the PD group was significantly less than that in the LITUS group.

**FIGURE 6:**
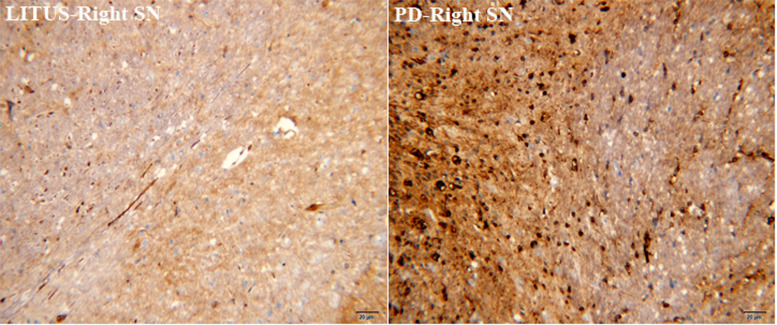
Iron staining showed that the number of iron+ cells in the PD group was significantly more than that in the LITUS group.

As shown in Figures 5, 6, the number of GDNF+-stained cells in the SN of the LITUS treatment group was significantly higher compared with the hemi-PD rat group. The mean staining scores for rats in the LITUS group were all between 3 and 4, while the scores were 2 and 3 in the hemi-PD rat group. The iron staining revealed that the iron staining+ cells in the SN of the LITUS group was weaker compared with the hemi-PD rat group. The mean staining scores for rats in the LITUS group were all between 2 and 4, while the scores were 3 and 5 in the hemi-PD rat group (see Supplementary Table 4 for details). Supplementary Table 5 shows the number of three kinds of immunohistochemical positive cells in the right SN of two groups of rats under high-power vision.

## Discussion

As a neurodegenerative disease, the course of PD includes loss or degeneration of dopamine neurons, microglial activation, and proliferation of astrocytes in the SN (Armstrong and Okun, 2020). 6-OHDA is a drug that is commonly used to create animal models of PD (Perlbarg et al., 2018). 6-OHDA is a selective chemical-damaging agent for DA neurons. When injected into the SN of rats, it can be actively absorbed into cells by the membrane transporters of DA neurons and oxidized to generate neurotoxic substances, which cause DA neurons to denature and die (Chi et al., 2019). Previous investigations have shown that the PD rat model created using 6-OHDA was more suitable for the study of early PD pathological changes (Liu L.X. et al., 2017). Therefore, we chose this model to study the early manifestations of PD as well as the early therapeutic effects of LITUS. Studies also have revealed that the number of TH+ cells in the SN decreased on the third day after the 6-OHDA injection, while the microglia and astrocytes proliferated to varying degrees, with the numbers peaking on the fourteenth day (Guiney et al., 2017). Thus, TH staining is a specific staining method for diagnosis of PD (Wakabayashi et al., 1990).

However, currently, most methods used to monitor the pathological changes in the brain rely on histology, which is invasive (Wakabayashi et al., 1990). Some researchers have used new non-invasive techniques, including DTI and T2^∗^WI to monitor the pathological changes in the SN of hemi-PD rats (Liu L.X. et al., 2017; Fang et al., 2018). Previous studies showed that the FA values in hemi-PD rats gradually increased, and T2^∗^ values gradually decreased (Fang et al., 2018), and that the pathological changes in the SN of hemi-PD rats can be monitored by these two parameters (Liu L.X. et al., 2017; Fang et al., 2018). Our experiments showed that FA first decreased then increased, while T2^∗^ first increased then decreased. Previous research results showed that FA value continues to rise; however, our experimental results are slightly different from previous studies, which might be due to the shorter intervals between scans and more comprehensive data collection. Our results also demonstrated that the FA value decreased significantly in the first week after the surgery compared with the FA value before the surgery, and this difference was statistically significant. After 6-OHDA injection, the number of dopaminergic neurons in the SN decreased significantly, which might have resulted in the uncomplicated neural structure in SN and the decrease of FA value. After the fifth week, the dopaminergic neuron damage was severe, and glia cell proliferation was observed. The structure of the SN neurons was disordered, and the FA value increased decidedly (Fang et al., 2018). However, the T2^∗^ value decreased in the fifth and sixth weeks, which might be related to the deposition of iron in the SN. Iron could have cause a gradual process of aggravation and resulted in increased change in T2^∗^ in the later stages of PD (Pietracupa et al., 2017). Therefore, this resulted in increased FA and decreased T2^∗^ values. Moreover, our results showed that TH staining in the hemi-PD rats was significantly lower than in the LITUS-treated rats, the number of GDNF-positive cells in the hemi-PD rats was significantly higher compared with the LITUS-treated rats, and the number of cells positive for iron staining was also significantly higher compared with normal rats. These staining results were consistent with the signal changes seen with MRI.

Compared with the hemi-PD rat group, the FA value of the LITUS rats increased significantly in the first week after treatment. However, there was no significant difference in the FA value before model induction. This observation suggested that LITUS had a protective effect on DA neurons and can reduce damage to the neurons. The specific protective mechanism of LITUS may be as follows. 6-OHDA was injected into the SN and taken directly into the DA neurons or indirectly through membrane transporters located on the cell bodies (Perlbarg et al., 2018). The 6-OHDA decreased the electron transfer efficiency of the mitochondrial chain in the DA neurons that resulted in damaged mitochondrial function and caused excessive ROS production, resulting in oxidative stress (Puspita et al., 2017). The persistent oxidative stress further aggravated mitochondrial dysfunction, resulting in apoptosis through the mitochondrial pathway (Roy et al., 2001). These results showed that the stability of the mitochondrial membrane potential was a prerequisite for maintaining normal mitochondrial function, and the decreased membrane potential led to mitochondrial dysfunction, which was an important feature of early apoptosis (Zamzami et al., 2005). Another study showed that LITUS could slow the depolarization of mitochondrial membrane potential that was induced by exposure to toxic substances and inhibit cell apoptosis (Fang et al., 2017). This effect of LITUS could occur because LITUS restored the mitochondrial membrane potential and ensured normal mitochondrial function that improved the oxidative stress induced by toxic substances and inhibited cell apoptosis in the dopaminergic neurons (Karmacharya et al., 2017).

In the fifth and sixth weeks, the FA value for LITUS rats was significantly lower compared with the hemi-PD rats. One possible mechanism was that the LITUS treatment resulted in increased stimulation of gliosis, and the increase in microglia promoted increased endogenous GDNF (Tang et al., 2017). Studies had shown that GDNF level in brain is definitely related to PD, and some researchers had used GDNF to treat PD patients (Allen et al., 2013). Another mechanism could be that LITUS slowly opened the blood–brain barrier (BBB) and promoted exogenous GDNF to enter the brain (Fan et al., 2016; Mead et al., 2017). GDNF promotes glial hyperplasia, which could replace the loss of glia caused by DA damage (Terse et al., 2020). Because cell proliferation was a chronic process, LITUS could reduce the anisotropy of the SN in the late stage of PD. Immunohistochemistry showed that the number of GDNF+ cells in the LITUS rats was significantly higher compared with the hemi-PD rats, which indicated increased astrocytes in the brain.

It is known that iron deposition gradually increases in PD and that LITUS can reduce iron deposition (Fang et al., 2018). We observed similar results in our study in that the significant differences in iron deposition only occurred in the later stages of the experiment. Our results show that the T2^∗^ value of the LITUS group was significantly higher than that of the PD group in the fifth and sixth weeks. Considering that LITUS can inhibit iron deposition in the SN of hemi-PD rats, we considered whether the mechanism might be twofold. On the one hand, some studies have shown that the SN in PD patients was in a low perfusion state (Wei et al., 2016). Low SN perfusion could cause decreased blood oxygen levels in the brain (Wei et al., 2016). Under hypoxic conditions, the extracellular electrolyte balance would be lost and the pH value would drop (Timofeev et al., 2013). When extracellular fluid is acidic, iron in ferritin is released in the form of Fe^2+^ through interaction with excessive superoxide free radicals and ascorbate (Abduljalil et al., 2003; Haacke et al., 2005). Thus, numerous studies support the possibility that LITUS could significantly improve brain microcirculation and reduce this effect (Yuan et al., 2019, 2020).

On the other hand, DA in the SN can produce neuromelanin granules, which bind with Fe^2+^ and block neuronal injury caused by Fe^2+^ (Li et al., 2012). However, when PD occurs, DA neurons were injured, which reduced the secretion of neuromelanin granules and released the loosely bound Fe^2+^, leading to Fe^2+^ deposition (Double and Halliday, 2006). As mentioned above, LITUS could protect DA neurons from damage and inhibit Fe^2+^ deposition. Because both mechanisms were evident later in the disease course, they also were associated with changes in T2^∗^ that we observed in the fifth and sixth weeks. Iron hematoxylin staining showed that the number of positive cells in the LITUS group was significantly less compared with the PD group, thus providing indirect support for this mechanism.

## Conclusion

LITUS is a non-invasive treatment method that could play a therapeutic role in hemi-PD rats by inhibiting dopamine neuron loss, promoting glial cell proliferation, and inhibiting iron ion deposition. Thus, LITUS is an effective treatment for PD. It is possible to closely monitor various pathological changes in the SN region by using the sensitive monitoring methods of DTI and T2^∗^WI, especially DTI. Pathological changes in the SN of hemi-PD rats can be monitored as soon as 1 week after disease. However, this study had some limitations. The experimental sample size was small, and animal experiments do not always closely mimic human clinical conditions. We hope that in future experiments, after obtaining appropriate ethics committee approval and increasing the experimental sample size, human studies can be carried out to obtain *in vivo* experimental data to provide a new non-invasive and effective treatment for PD patients and provide the MRI technology to assess this therapeutic effect to clinical doctors.

## Data Availability Statement

All datasets generated for this study are included in the article/Supplementary Material, further inquiries can be directed to the corresponding author/s.

## Ethics Statement

The experiments were conducted in accordance with national guidelines for the use of experimental animals. All experimental protocols were approved by the Ethics Committee of Qinhuangdao Municipal No. 1 Hospital.

## Author Contributions

YD, DL, YZ, and LL contributed to the design of the study and the development of the study protocol. YZ coordinated the study. ZW and LL performed the systematic review, including data collection, and data analysis. YD drafted the first version of the manuscript. All authors contributed to data interpretation, manuscript drafting, and review.

## Conflict of Interest

The authors declare that the research was conducted in the absence of any commercial or financial relationships that could be construed as a potential conflict of interest.
