# Supplementary material for: Assessment of Neuroprotective Effects of Low-Intensity Transcranial Ultrasound Stimulation in a Parkinson’s Disease Rat Model by Fractional Anisotropy and Relaxation Time T2∗ Value
Source: Front Neurosci. 2021 Feb 9;15:590354. doi: 10.3389/fnins.2021.590354 (PMC7900573; doi:10.3389/fnins.2021.590354)
Supplement: Supplementary file 1 [file Table_1.docx]

Table 1. Detailed data of rotation experiment

|  | 1^st^ week | | 2^nd^ week | | 3^rd^ week | | 4^th^ week | | 5^th^ week | | 6^th^ week | |
| --- | --- | --- | --- | --- | --- | --- | --- | --- | --- | --- | --- | --- |
| Case | PD | LITUS | PD | LITUS | PD | LITUS | PD | LITUS | PD | LITUS | PD | LITUS |
| 1 | 8 | 7 | 8 | 8 | 9 | 8 | 8 | 7 | 8 | 8 | 9 | 8 |
| 2 | 8 | 9 | 8 | 9 | 8 | 9 | 8 | 9 | 8 | 9 | 8 | 9 |
| 3 | 7 | 8 | 7 | 8 | 7 | 10 | 9 | 8 | 10 | 7 | 7 | 9 |
| 4 | 9 | 9 | 8 | 9 | 8 | 9 | 9 | 9 | 8 | 9 | 8 | 9 |
| 5 | 8 | 10 | 8 | 9 | 8 | 9 | 8 | 10 | 8 | 9 | 8 | 9 |
| 6 | 9 | 8 | 9 | 8 | 9 | 8 | 9 | 8 | 9 | 8 | 9 | 8 |
| 7 | 7 | 9 | 7 | 9 | 9 | 9 | 7 | 9 | 7 | 9 | 9 | 9 |
| 8 | 8 | 9 | 8 | 9 | 8 | 9 | 8 | 9 | 8 | 10 | 8 | 9 |
| 9 | 9 | 8 | 9 | 8 | 9 | 8 | 9 | 8 | 9 | 8 | 8 | 8 |
| 10 | 10 | 7 | 10 | 7 | 9 | 7 | 10 | 8 | 8 | 7 | 9 | 7 |
| mean±SD/min | 8.3±0.95 | 8.4±0.97 | 8.2±0.92 | 8.4±0.70 | 8.4±0.70 | 8.6±0.84 | 8.5±0.85 | 8.5±0.85 | 8.3±0.82 | 8.4±0.97 | 8.3±0.67 | 8.5±0.71 |

This table shows the weekly number of Apomorphine-induced rotations of rats in each group.
