# Supplementary material for: Assessment of Neuroprotective Effects of Low-Intensity Transcranial Ultrasound Stimulation in a Parkinson’s Disease Rat Model by Fractional Anisotropy and Relaxation Time T2∗ Value
Source: Front Neurosci. 2021 Feb 9;15:590354. doi: 10.3389/fnins.2021.590354 (PMC7900573; doi:10.3389/fnins.2021.590354)
Supplement: Supplementary file 2 [file Table_2.docx]

Table 2. Detailed comparison of FA values in the right SN between the two groups.

|  | LITUS | PD | t value | P value |
| --- | --- | --- | --- | --- |
| Pre-surgery | 0.303±0.016 | 0.295±0.024 | 0.286 | 0.779 |
| 1^st^ week | 0.340±0.032 | 0.214±0.027 | 2.864 | 0.011* |
| 2^nd^ week | 0.324±0.030 | 0.249±0.041 | 1.526 | 0.147 |
| 3^rd^ week | 0.298±0.037 | 0.306±0.026 | 0.174 | 0.864 |
| 4^th^ week | 0.326±0.032 | 0.314±0.033 | 0.264 | 0.795 |
| 5^th^ week | 0.290±0.037 | 0.405±0.027 | 2.385 | 0.030* |
| 6^th^ week | 0.299±0.021 | 0.525±0.028 | 6.620 | < 0.0001* |

* P value was less than 0.05, the difference was statistically significant.
