# Supplementary material for: Assessment of Neuroprotective Effects of Low-Intensity Transcranial Ultrasound Stimulation in a Parkinson’s Disease Rat Model by Fractional Anisotropy and Relaxation Time T2∗ Value
Source: Front Neurosci. 2021 Feb 9;15:590354. doi: 10.3389/fnins.2021.590354 (PMC7900573; doi:10.3389/fnins.2021.590354)
Supplement: Supplementary file 3 [file Table_3.docx]

Table 3. Detailed comparison of T2^*^ values in the right SN between the two groups.

|  | LITUS | PD | t value | P value |
| --- | --- | --- | --- | --- |
| Pre-surgery | 11.50±0.528 | 10.75±0.507 | 1.009 | 0.328 |
| 1^st^ week | 12.50±1.206 | 9.51±0.851 | 1.926 | 0.072 |
| 2^nd^ week | 13.76±0.772 | 12.50±0.886 | 1.076 | 0.298 |
| 3^rd^ week | 14.38±0.786 | 13.25±0.687 | 1.058 | 0.306 |
| 4^th^ week | 12.60±0.736 | 11.00±0.782 | 1.482 | 0.158 |
| 5^th^ week | 12.17±0.826 | 7.55±0.824 | 3.907 | 0.001* |
| 6^th^ week | 11.75±0.615 | 7.55±0.849 | 4.104 | 0.0008* |

* P value was less than 0.05, the difference was statistically significant.
