# Supplementary material for: Assessment of Neuroprotective Effects of Low-Intensity Transcranial Ultrasound Stimulation in a Parkinson’s Disease Rat Model by Fractional Anisotropy and Relaxation Time T2∗ Value
Source: Front Neurosci. 2021 Feb 9;15:590354. doi: 10.3389/fnins.2021.590354 (PMC7900573; doi:10.3389/fnins.2021.590354)
Supplement: Supplementary file 4 [file Table_4.docx]

Table 4 Staining scores of the 20 rats in the two groups

| Case | Groups | Grading scores | | |
| --- | --- | --- | --- | --- |
|  |  | TH | GDNF | iron |
| 1 | LITUS | 4 | 3 | 2 |
| 2 | LITUS | 5 | 4 | 3 |
| 3 | LITUS | 5 | 4 | 2 |
| 4 | LITUS | 5 | 4 | 4 |
| 5 | LITUS | 4 | 3 | 2 |
| 6 | LITUS | 4 | 4 | 3 |
| 7 | LITUS | 5 | 3 | 2 |
| 8 | LITUS | 4 | 4 | 2 |
| 9 | LITUS | 5 | 4 | 3 |
| 10 | LITUS | 5 | 4 | 2 |
| 1 | PD | 2 | 2 | 3 |
| 2 | PD | 3 | 3 | 4 |
| 3 | PD | 2 | 3 | 3 |
| 4 | PD | 2 | 2 | 5 |
| 5 | PD | 1 | 2 | 5 |
| 6 | PD | 2 | 3 | 4 |
| 7 | PD | 2 | 2 | 3 |
| 8 | PD | 1 | 2 | 3 |
| 9 | PD | 2 | 3 | 4 |
| 10 | PD | 1 | 2 | 4 |

The grading scores of the rats of all rats in two groups were rounded up to the nearest whole number.
