# Supplementary material for: Assessment of Neuroprotective Effects of Low-Intensity Transcranial Ultrasound Stimulation in a Parkinson’s Disease Rat Model by Fractional Anisotropy and Relaxation Time T2∗ Value
Source: Front Neurosci. 2021 Feb 9;15:590354. doi: 10.3389/fnins.2021.590354 (PMC7900573; doi:10.3389/fnins.2021.590354)
Supplement: Supplementary file 5 [file Table_5.docx]

Table 5. Immunohistochemical positive cell results

| Group | Case | TH+ | GDNF+ | iron+ |
| --- | --- | --- | --- | --- |
| LITUS | 1 | 102 | 82 | 23 |
|  | 2 | 121 | 78 | 18 |
|  | 3 | 119 | 69 | 21 |
|  | 4 | 89 | 74 | 17 |
|  | 5 | 97 | 81 | 31 |
|  | 6 | 94 | 77 | 18 |
|  | 7 | 108 | 68 | 22 |
|  | 8 | 92 | 74 | 26 |
|  | 9 | 112 | 84 | 24 |
|  | 10 | 105 | 80 | 32 |
| PD | 1 | 22 | 28 | 189 |
|  | 2 | 32 | 33 | 201 |
|  | 3 | 55 | 32 | 178 |
|  | 4 | 48 | 21 | 169 |
|  | 5 | 52 | 34 | 176 |
|  | 6 | 27 | 28 | 183 |
|  | 7 | 34 | 37 | 187 |
|  | 8 | 29 | 26 | 192 |
|  | 9 | 33 | 25 | 166 |
|  | 10 | 28 | 32 | 178 |

The Table showed the number of three kinds of immunohistochemical positive cells in the right SN of two groups of rats under high power vision.
